# Nicotinamide Mononucleotide Ameliorates Cellular Senescence and Inflammation Caused by Sodium Iodate in RPE

**DOI:** 10.1155/2022/5961123

**Published:** 2022-07-18

**Authors:** Chengda Ren, Chengyu Hu, Yan Wu, Tingting Li, Aiqi Zou, Donghui Yu, Tianyi Shen, Wenting Cai, Jing Yu

**Affiliations:** Department of Ophthalmology, Shanghai Tenth People's Hospital, School of Medicine, Tongji University, Shanghai 200072, China

## Abstract

Senescent cells have been demonstrated to have lower cellular NAD^+^ levels and are involved in the development of various age-related diseases, including age-related macular degeneration (AMD). Sodium iodate (NaIO_3_) has been primarily used as an oxidant to establish a model of dry AMD. Results of previous studies have showed that NaIO_3_ induced retinal tissue senescence *in vivo*. However, the role of NaIO_3_ and the mechanism by which it induces retinal pigment epithelium (RPE) senescence remains unknown. In this study, RPE cell senescence was confirmed to be potentially induced by NaIO_3_. The results showed that the number of senescence-associated-*β*-galactosidase (SA-*β*-gal-)-positive cells and the protein levels of p16 and p21 increased after NaIO_3_ treatment. Additionally, the senescent RPE cells underwent oxidative stress and NAD^+^ depletion. Furthermore, significant DNA damage and mitochondrial dysfunction were also detected in senescent RPE cells. The antioxidant N-acetylcysteine (NAC) could alleviate cellular senescence only by a minimal degree, whereas supplementation with nicotinamide mononucleotide (NMN) strongly ameliorated RPE senescence through the alleviation of DNA damage and the maintenance of mitochondrial function. The protective effects of NMN were demonstrated to rely on undisturbed Sirt1 signaling. Moreover, both the expression of senescence markers of RPE and subretinal inflammatory cell infiltration were decreased by NMN treatment *in vivo*. Our results indicate that RPE senescence induced by NaIO_3_ acquired several key features of AMD. More importantly, NMN may potentially be used to treat RPE senescence and senescence-associated pre-AMD changes by restoring the NAD^+^ levels in cells and tissues.

## 1. Introduction

Age-related macular degeneration (AMD) is one of the leading causes of irreversible blindness among the elderly individuals [1]. Late-stage AMD is present in two distinct forms: choroidal neovascularization and geographic atrophy. AMD is a multifactorial disease governed by both endogenous and exogenous risk factors. Among these, aging is the most crucial risk factor [2] because it causes additional stress to tissues. One of the hallmarks of an aged organism is the accumulation of senescent cells [3]. Senescence is characterized by an irreversible cell cycle arrest [4]. Even though senescence progression demonstrates potential positive effects on tissue repair, excessive accumulation of senescent cells can stimulate local inflammation, thus promoting the onset and development of multiple age-related diseases such as AMD [5, 6].

The retinal pigment epithelium (RPE) is a monolayer cellular barrier between the neural retina and the choroid region. The homeostasis of these organs depends on the physiological function of the RPE [7]. AMD affects both the outer retina and the choroid; therefore, the RPE plays an important role in the incidence of AMD. With aging, the RPE cells experience telomere erosion, accumulation of oxidative products, and prominent DNA damage [8, 9], as all of these factors induce the senescence of RPE. An increasing amount of evidence demonstrates that senescent RPE cells can accumulate on human AMD donor eyes [10–13]. Therefore, targeting cellular senescence of RPE cells may have beneficial effects on relieving the progression of AMD.

Sodium iodate (NaIO_3_) is a widely used compound to investigate the pathogenesis of dry AMD since it causes retinal degeneration in a dose- and time-dependent manner. Multiple studies have investigated the oxidative toxicity and cell death caused by NaIO_3_ [14–17]. However, an important feature of age-related diseases is that the tissues are constantly exposed to long-term and low-intensity stimulations. Therefore, determination of the nonlethal effects of NaIO_3_ is of great importance. Li and co-workers have reported that a low dose of NaIO_3_ causes retinal senescence through oxidative stress [18]. However, the role of NaIO_3_ in inducing RPE senescence has not been clarified thus fat.

NAD^+^ is one of the most important coenzymes involved in energy metabolism since it functions as a substrate for cyclic ADP-ribose hydrolase (CD38), poly-ADP-ribose polymerases (PARPs), and sirtuins [19]. NAD^+^ content decreases with aging in various types of tissues, including retina and RPE [20]. Recently, it has been reported that various retinal disease models also exhibited early decline in retinal NAD^+^ level [21]. Although direct administration of NAD^+^ is difficult, the precursors of NDA^+^ are more stable and can be administered more easily. Previous studies suggested that supplementation with NAD^+^ precursors such as nicotinamide mononucleotide (NMN) may potentially mitigate several age-related disorders [22, 23]. However, the extent of involvement of NMN and the mechanism by which NMN decrease the RPE senescence have not been clarified thus far. Here, we established a senescence model, which acquired several features of AMD. Additionally, our results showed that RPE senescence was mainly mediated by mitochondrial dysfunction. Moreover, we showed that NMN supplementation replenished the NAD^+^ levels in RPE cells and ameliorated RPE senescence and senescence-associated retinal inflammation. Furthermore, we demonstrated that an undisturbed Sirt1 signalling is required for NMN to exert its protective effects.

## 2. Materials and Methods

### 2.1. Reagents

The Ex527 was purchased from Beyotime (Beyotime, Shanghai, China), and the SRT1720 was obtained from AbMole (Abmole Bioscience Inc., Houston, TX, USA). The N-acetyl-l-cysteine (NAC) was purchased from MedChemExpress (MCE). The antibodies specific for p16 (10883-1-AP), TOM20 (11802-1-AP), PGC-1*α* (66369-1-Ig), F4/80 (28463-1-AP), and ZO-1 (21773-1-AP) were purchased from Proteintech (Proteintech Group, Inc., Rosemont, IL, USA). The antibodies specific for p21 (#37543), Sirt1 (#2493), AMPK*α* (#5831), phosphor-AMPK*α* (Thr172, #2535), total-Akt (#4685), phosphor-Akt (Ser473, #4060), mTOR (#2983), and phosphor-mTOR (Ser2448, #5536), *λ*-H2AX (#2577) was purchased from Cell Signaling Technology (Beverly, MA, USA). The antibodies specific for OPA-1 (sc-393296), MFN-1 (sc-166644), MFN-2 (sc-515647), Drp-1 (sc-271583), and Fis (sc-376447) were obtained from Santa Cruz Biotechnology (Santa Cruz, CA, USA). The antibody specific for CD11b (53-0112-82) was purchased from Thermo Fisher Scientific. The antibodies specific for *β*-actin (P60709), *β*-tubulin (M30109), and GAPDH (M20006) were purchased from Abmart Biotechnology (Abmart, Shanghai, China). The specific antibody of acetyl-p65 (ab19870) was purchased from Abcam (Abcam, Cambridge, UK). The secondary antibodies for western blot was purchased from LI-COR Biotechnology (LI-COR, Lincoln, NE, USA). The secondary antibodies for immunofluorescent analysis were purchased from Invitrogen Biotechnology (Thermo Fisher Scientific, Waltham, MA, USA).

### 2.2. Mice

C57BL/6J male mice (6-8 weeks old) were purchased from Beijing Vital River Laboratory Animal Technology (Beijing, China). The animal experiments were all performed according to the ARRIVE guidelines and the ARVO Statement for the Use of Animals in Ophthalmic and Vision. All animal experiments were authorized by the ethical committee of Shanghai Tenth People's Hospital. All animals were given free access to food and drinking water. Mice were housed in a pathogen-free room with constant temperature (22°C) under a 12 h light-dark cycle. NaIO_3_ (Sigma-Aldrich, San Francisco, CA, USA) was dissolved in sterile saline at the concentrations from 1 mg/ml to 8 mg/ml to ensure that each group of mice received the nearly same volume of solution. The solution was given as a single dose at the indicated concentration intraperitoneally. The control group received the same volume of vehicle. NMN (Hygieia Biotechnology, China) with a purity of over 99.8% was dissolved in sterile saline at the concentration of 20 mg/ml. Mice in the NMN group were intraperitoneally administrated with NMN (300 mg/kg/day) for 7 consecutive days. Mice in the NaIO_3_ group and the control group received intraperitoneal injection of the same amount of sterile saline for 7 consecutive days. Then, the mice were administrated with NaIO_3_ (10 mg/kg) and sterile saline, respectively. After that, mice in the NMN group continued receiving NMN treatment for 7 days, while mice in the NaIO_3_ and the control group received sterile saline treatment for 7 days.

### 2.3. Cell Culture

Adult human RPE cell line ARPE-19 cell was purchased from MEISENCTCC company (Hangzhou, China). All cells used in our experiments were between passage 6 and 20. DMEM/F12 culture media (Thermo Fisher Scientific) with 10% fetal bovine serum (FBS, Gibco, Carlsbad, CA, USA), 100 U/mL penicillin and 100 mg/mL streptomycin was used in cell culture. All cells were incubated at 37°C under an atmosphere of 5% CO_2_. For further analysis, cells were seeded in 6-, 12-, 24-, or 96-well plate as needed, and the FBS was reduced to 1% as reported in a previous study [8]. Cells were seeded at 10,000/cm^2^ or 20,000/cm^2^ for immunofluorescent assays and other assays, respectively.

### 2.4. Cell Viability Assay

After the treatment, the cell viability was measured with CCK-8 kit (Yeasen, Shanghai, China) according to the manufacturer's protocol and then was detected with a microplate reader (BioTek, VT, USA).

### 2.5. Senescence-Associated-*β*-Gal Staining

The senescence-associated-*β*-galactosidase staining kit (Beyotime) was used to detect the senescent status of ARPE-19 cells as well as ocular tissues. Experiments were conducted in accordance with the manufacturer's protocol. Briefly, cells and frozen sections were washed in PBS and fixed for 15 min in 4% paraformaldehyde. Then, they were washed in PBS and incubated with SA-*β*-gal staining solution at 37°C overnight. The stained cells and tissue sections were examined under phase-contrast microscopy (Nikon Corporation, Tokyo, Japan).

### 2.6. Western Blot Analysis

After treatment, cells were washed with precooled PBS and lysed with RIPA buffer with protease inhibitor (Protease Inhibitor Cocktail, Sigma-Aldrich), 50 mM NaF, 100 *μ*M Na3VO_4_ (Service Bioscience, Wuhan, China), and 1 mM PMSF (Beyotime, Shanghai, China). All subsequent protein extracting procedures were performed in a standard manner. A total of 30 *μ*g of protein extracts were loaded on per channel of 7.5%-12.5% polyacrylamide gels. After electrophoresis, proteins were transferred to nitrocellulose membrane (Whatman, UK). Then, the membrane was blocked with 5% nonfat milk or 5% BSA. After blocking, the membrane was incubated with primary antibody at 4°C overnight and then incubated with specific secondary antibody at room temperature for 1 h. At last, the protein bands were scanned by Odyssey (LI-COR). The band intensity was analyzed by Image Studio System (Version 5.2.5).

### 2.7. Quantitative PCR

After treatment, total RNA was extracted by EZ-press RNA purification kit (Roseville, MN, USA) and RNA concentration was determined with NanoDrop 3300 (Thermo Fisher Scientific). cDNA was synthesized from 1 *μ*g of total RNA using HiScript III 1st strand cDNA synthesis kit (Vazyme, Nanjing, China). The qPCR analysis was performed using ChamQ Universal SYBR qPCR Master Mix (Vazyme). The contents of different mRNA targets in different groups were calculated by *^ΔΔ^*Ct method. Primers were synthesized by Sangon Biotech (Sangon Biotech, Shanghai, China). Primers used in the experiments were as follows: human-p16-F (5′-GGGGGCACCAGAGGCAGT-3′), human-p16-R (5′-GGTTGTGGCGGGGGCAGTT-3′), human-p21-F (5′-TCCTCATCCCGTGTTCTCCT-3′), human-p21-R (5′-CACCCTGCCCAACCTTAGAG-3′), human-GAPDH-F (5′-GGACCTGACCTGCCGTCTAGAA-3′), human-GAPDH-R (5′-GGTGTCGCTGTTGAAGTCAGAG-3′).

### 2.8. Cell Immunofluorescent Assay

Cells were harvested and washed with PBS and then fixed with 4% paraformaldehyde for 15 min. After that, antigen blocking was conducted at room temperature for 1 h with 5% BSA added with 0.1% Triton X-100. Different primary antibodies were applied to perform incubation at 4°C overnight. Then, cells were incubated with fluorescent secondary antibody. Before mounting with ProLong antifade reagent (Thermo Fisher Scientific), cells were stained with DAPI (Sigma-Aldrich) for 10 min. The pictures were captured by an upright fluorescence microscopy (Leica Microsystems, Wetzlar, Germany) or the LSM 980 Airyscan SR microscopy (Carl Zeiss, Micro Imaging GmbH, Jena, Germany) as needed.

### 2.9. Measurement of Cytosolic ROS

Cytosolic ROS level was detected using H2DCFDA (Sigma-Aldrich). Briefly, after treatment, cells were washed with PBS and incubated with 10 *μ*M H2DCFDA probe for 30 min. After incubation, cells prepared for quantitative analysis were washed with PBS and trypsinized and collected for flow cytometry analysis.

### 2.10. Determination of the Intracellular ATP Level

Intracellular ATP level was measured through the ATP assay kit (Beyotime), and the procedure was conducted in accordance with the manufacturer's instruction. Briefly, after lysis, the lysate was centrifuged and the supernatant was retained. Then, 100 *μ*L working solution and 20 *μ*L sample were added onto the testing well. The mixture was detected by a microplate reader (BioTek, VT, USA).

### 2.11. Nicotinamide Adenine Dinucleotide Measurement

The cellular and tissue NAD^+^ level was determined with a NAD/NADH assay kit (Abcam) according to the manufacturer's instruction.

### 2.12. Mitochondrial Membrane Potential Assay

The mitochondrial membrane potential was determined using JC-1 mitochondrial membrane potential assay kit (Yeasen) according to the manufacturer's protocol. After staining, the images were taken with an inverted fluorescence microscope (Nikon Corporation).

### 2.13. Determination of Mitochondrial Mass

The mitochondrial mass was determined by the protein level of TOM20 and mito-tracker staining. Mito-Tracker Green (Beyotime, Shanghai, China) was diluted in accordance with the manufacture's protocol. Cells were incubated with Mito-Tracker Green for 45 min and washed with preheated PBS. Then, cells were incubated with Hoechst (Beyotime, Shanghai, China) for 15 min. The results were obtained using an inverted fluorescence microscope (Nikon Corporation) and flow cytometry, respectively.

### 2.14. Immunofluorescence of RPE Flatmount

After drug treatment, the eyes were enucleated and fixed in 4% paraformaldehyde for 30 min, after which the retina were carefully detached from the RPE-choroid-sclera complex under a microscope. Then, the RPE mounts were cut into four sections conducted standard immunofluorescence staining procedure with the primary antibodies against ZO-1 and CD11b. Photos were taken by an upright fluorescence microscopy (Leica Microsystems).

### 2.15. Immunofluorescence of Retinal Slice

After drug treatment, the mice were sacrificed, and the eyes were immersed in 4% paraformaldehyde for 24 h to be fixed. Then, the eyes were embedded in paraffin and cut into 5 *μ*m serially sections. After that, the sections were dewaxing and hydrated. Citric acid was used for antigen repairing. Then, the sections were blocked with 5% BSA and 0.5% TritonX-100. Primary antibody against F4/80 and secondary antibody were incubated successively. Sections were stained with DAPI for 10 min before mounting. Photos were taken by an upright fluorescence microscopy (Leica Microsystems).

### 2.16. Hematoxylin and Eosin Staining

The mice were sacrificed after drug treatment, and the eyes were immersed in 4% paraformaldehyde for 24 h to be fixed. After fixation, paraffin-embedded serially sections of 3 *μ*m were cut carefully and stained with hematoxylin-eosin (H&E). Photos of the sections were taken using a light microscope (Leica Microsystems). Changes of the thickness of retinal outer nuclear layer (ONL) were used for quantitatively evaluating histological alterations.

### 2.17. Statistical Analysis

Each experiment was repeated at least thrice. Graphpad Prism 9 was used to perform statistical analyses. All data was expressed as the mean ± SD, statistical differences were determined by Student's *t*-test for comparison between two groups, and ANOVA followed by Dunnett's multiple comparison test for comparison among three or more groups. *p* < 0.05 was considered to be statistically significant.

## 3. Results

### 3.1. Establishment of a Model of RPE Cellular Senescence

**Figure 1:**
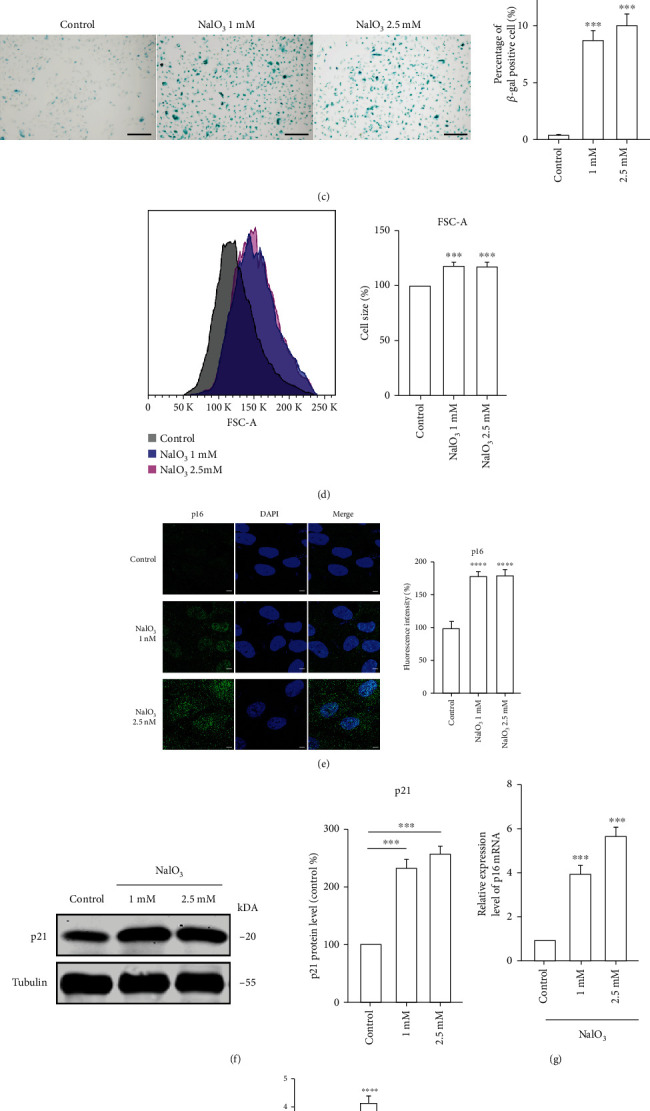
(a–b) ARPE-19 cells were treated with different concentrations of NaIO_3_ for 24 h or 48 h. Cell viability was analyzed by CCK-8 assay (*n* = 5). (c–h) ARPE-19 cells were treated with 1 and 2.5 mM NaIO_3_ for 48 h. (c) The representative images of SA-*β*-gal staining in ARPE-19 cells; scale bar = 200 *μ*m (*n* = 3). (d) Relative cell size distribution in ARPE-19 cells. *X* axis is FSC-A, which reflects cell size (*n* = 3). (e) p16 immunostaining in ARPE-19 cells; scale bar = 10 *μ*m (*n* = 3). (f) Proteins were extracted from ARPE-19 cells. p21 protein levels were evaluated by western blot and *β*-tubulin was used as a loading control (*n* = 3). (g–h) Quantification of p16 and p21 mRNA expression in ARPE-19 cells (*n* = 3). GAPDH was used as a loading control. Data represent mean ± SD of at least three independent experiments, ∗*p* < 0.05, ∗∗*p* < 0.01, ∗∗∗∗*p* < 0.0001, compared versus control.

We first established a senescent cell model utilizing a nonlethal NaIO_3_ stimulus. We monitored the cell viability after treatment with varying concentrations of NaIO_3_ at 24-h and 48-h intervals. The cell viability decreased by approximately 50% after exposure to 5 mM NaIO_3_ (Figures 1(a) and 1(b)), indicating that concentrations below 5 mM were suitable for establishing the model. Then, we detected the expression of several senescence markers including senescence-associated-*β*-Gal (SA-*β*-gal), cell size, and expression levels of p16 and p21. Results of the SA-*β*-gal demonstrated that nearly 10% cells became senescent after the treatment (Figure 1(c)). Additionally, the cell size increased by 20% relative to the control group (Figure 1(d)). Moreover, the protein and mRNA levels of p16 and p21 increased after exposure to 1 and 2.5 mM NaIO_3_ (Figures 1(e)–1(h)).

### 3.2. Oxidative Stress Is Involved in the NaIO_3_-Induced ARPE-19 Cell Senescence

**Figure 2:**
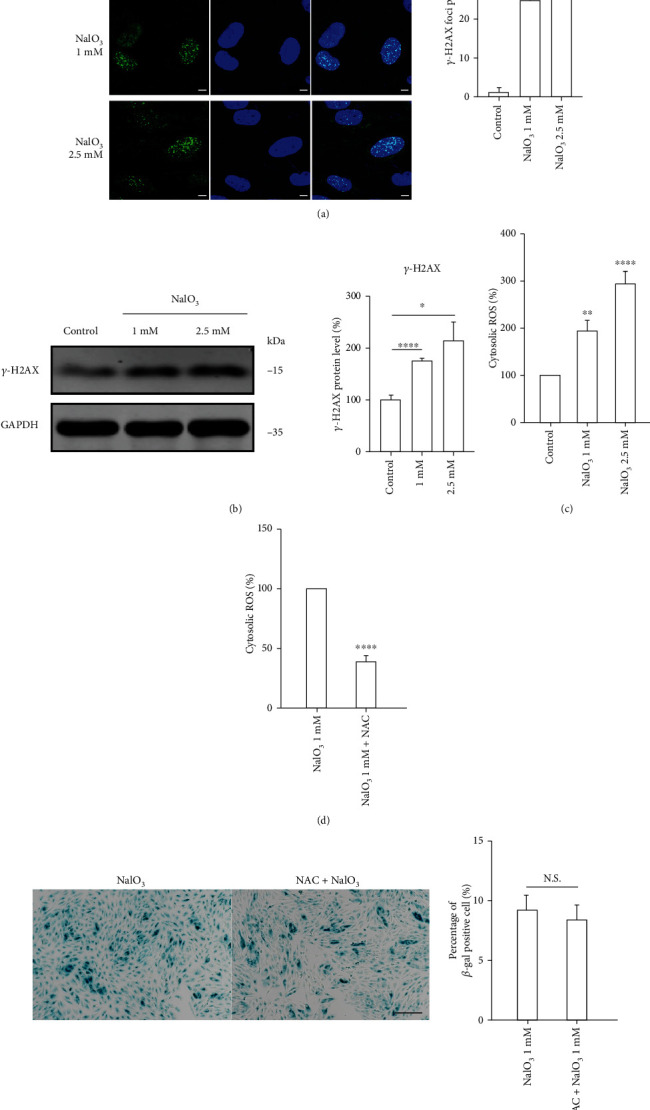
(a) The representative images and the statistical results of immunofluorescent analysis of *λ*-H2AX foci in ARPE-19 treated with 1 and 2.5 mM NaIO_3_ for 48 h; scale bar = 10 *μ*m (*n* = 3). (b) The western blot and the statistical results of levels of *λ*-H2AX protein in NaIO_3_ stimulated ARPE-19 cells (*n* = 3). (c) Cells were treated with NaIO_3_ for 3 h. Cytosolic ROS levels were determined by flow cytometry (*n* = 3). (d) Cells were pretreated with 10 mM NAC for 24 prior to NaIO_3_ treatment for 3 h. Cytosolic ROS levels were determined by flow cytometry (*n* = 3). (e) The representative images and the quantitative results of SA-*β*-gal staining in ARPE-19 cells treated as indicated; scale bar = 200 *μ*m (*n* = 3). Data represent mean ± SD of at least three independent experiments, ∗*p* < 0.05, ∗∗*p* < 0.01, ∗∗∗*p* < 0.001, ∗∗∗∗*p* < 0.0001, compared versus control or between the indicated groups.

After successfully inducing RPE senescence, we investigated the mechanism underlying the development of senescence. We observed increased expression of *λ*-H2AX, a marker of DNA double-strand breaks, in cells treated with NaIO_3_. The expression of this marker indicates that the DNA damage response (DDR) may contribute to RPE senescence (Figures 2(a) and 2(b)). Since NaIO_3_ is an oxidant, we speculated that generation of ROS caused the excessive DNA damage induced by NaIO_3_. Therefore, we measured the ROS levels following NaIO_3_ treatment. The results showed that the ROS levels of the NaIO_3_-treated group were ~2- to 3-fold relative to the control group (Figure 2(c)). Treatment with the antioxidant NAC significantly decreased the ROS levels in cells treated with NaIO_3_ (Figure 2(d)) but failed to prevent the RPE cells from entering senescence (Figure 2(e)). Therefore, an essential factor apart from oxidative stress may be involved in the control of cellular senescence.

### 3.3. Dysfunctional Mitochondria Accumulate and Are Hyperfused in Senescent ARPE-19 Cells

**Figure 3:**
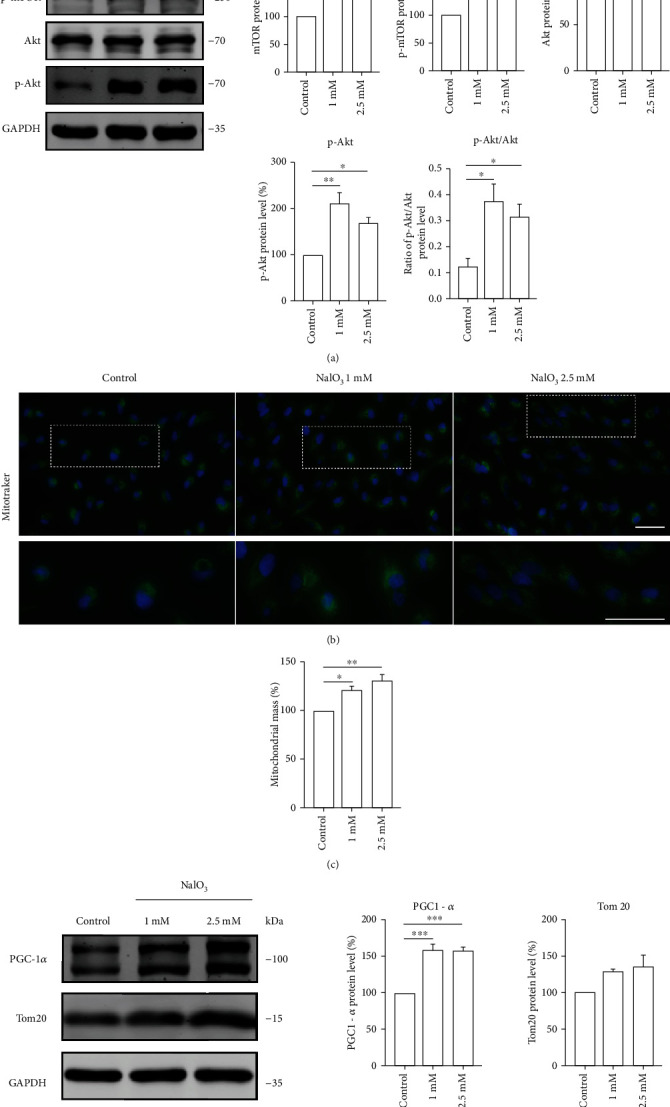
(a–d) ARPE-19 cells were threaten with 1 and 2.5 mM NaIO_3_ for 48 h. (a) The protein levels and the statistical results of indicated proteins in ARPE-19 cells. GAPDH was used as an internal control (*n* = 3). (b–c) The representative microscopy images of mito-tracker staining in ARPE-19 cells. The results were quantified by flow cytometry; scale bar = 50 *μ*m (*n* = 3). (d) Protein levels of PGC-1*α* (*n* = 3) and Tom20 (*n* = 3) in ARPE-19 cells. GAPDH was used as a loading control. Data represent mean ± SD of at least three independent experiments, ∗*p* < 0.05, ∗∗*p* < 0.01, ∗∗∗*p* < 0.001, ∗∗∗∗*p* < 0.0001, compared versus control.

The Akt/mTOR signaling was reported to integrate signals from DDR towards mitochondrial biogenesis and is involved in senescence [24]. We detected the expression of Akt-mTOR signalling pathway-related genes and found that they were strongly activated in senescent RPE cells (Figure 3(a)). Subsequently, we used the mito-tracker green staining to verify this hypothesis and evaluate the mitochondrial biomass. The increased fluorescence intensity indicated that senescent cells had a 20% higher mitochondrial content (Figures 3(b) and 3(c)). Moreover, we observed an upregulation in the levels of PGC-1*α* and Tom20 proteins. Our results showed that NaIO_3_ treatment induced mitochondrial biogenesis (Figure 3(d)).

**Figure 4:**
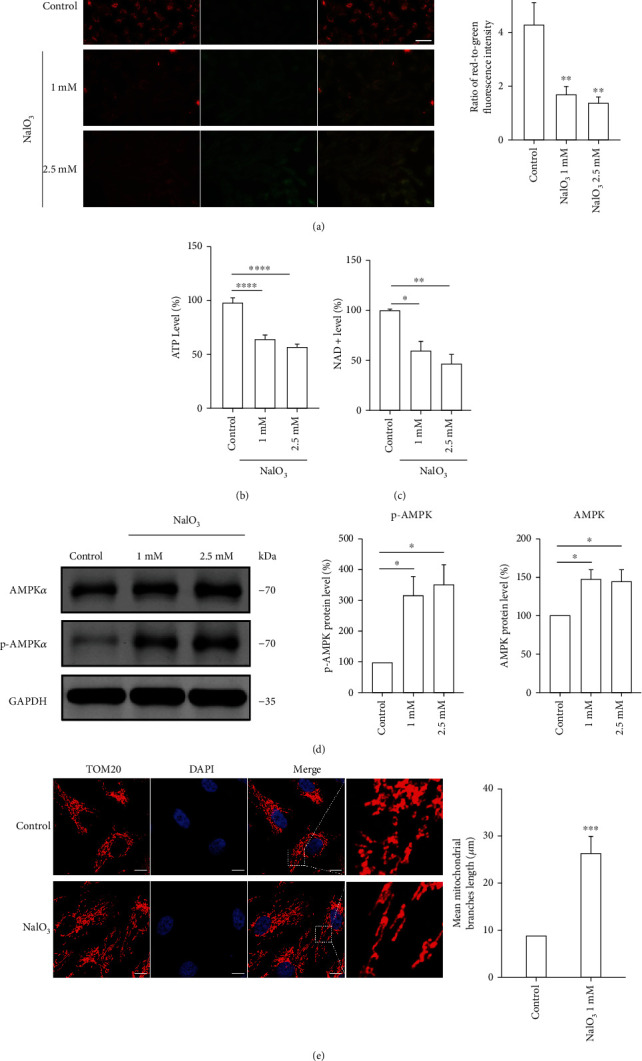
(a–d) ARPE-19 cells were treated with 1 and 2.5 mM NaIO_3_ for 48 h. (a) Mitochondrial membrane potential (MMP) in ARPE-19 cells was demonstrated by JC-1 staining, and the results were obtained by fluorescent microscopy. The red-to-green fluorescence intensity was used to quantitatively evaluate the mitochondrial potential; scale bar = 50 *μ*m (*n* = 3). (b) Cellular ATP levels in ARPE-19 cells (*n* = 5). (c) NAD^+^ levels in ARPE-19 cells (*n* = 3). (d) Expression of AMPK*α* and p-AMPK*α* proteins in ARPE-19 cells. (e) Immunofluorescence assay of mitochondrial outer membrane marker Tom20 was performed by confocal microscope. Cells were treated with 1 mM NaIO_3_ for 48 hr; scale bar = 10 *μ*m (*n* = 3) The mitochondrial branches length were measured by ImageJ. Data represent mean ± SD of at least three independent experiments, ∗*p* < 0.05, ∗∗*p* < 0.01, ∗∗∗*p* < 0.001, ∗∗∗∗*p* < 0.0001, compared versus control.

In addition to its function as an oxidant, NaIO_3_ was also reported to inhibit mitochondrial metabolic enzymes directly [25, 26], and mitochondrial dysfunction plays a prominent role in the pathogenesis of AMD [27]. Therefore, it is important to explore the functional changes in the mitochondria in NaIO_3_-treated cells. In our study, we observed a significant decrease in the mitochondrial membrane potential (MMP) of cells treated with different concentrations of NaIO_3_ (Figure 4(a)). Similarly, cellular ATP and NAD^+^ levels decreased after NaIO_3_ treatment (Figures 4(b) and 4(c)). Previous studies showed that AMPK is regulated by the AMP/ATP ratio and plays an important role in the resistance to cellular stress. We confirmed that AMPK signalling was activated due to the decrease in the ATP levels (Figure 4(d)). The toxic effects of NaIO_3_ are highly correlated to its concentration because high concentrations of NaIO_3_ induced mitochondrial fission in ARPE-19 cells [28]. However, our results showed that the mitochondria in senescent cells had a more tubular structure (Figure 4(e)). Previous studies have also shown that PGAM5^−/−^ ARPE-19 cells automatically enter the senescent state with hyperfused mitochondria [29]. Thus, we extrapolated that the mitochondrial hyperfusion was correlated with RPE senescence.

### 3.4. NMN Ameliorates Cellular Senescence by Improving the Mitochondrial Function

**Figure 5:**
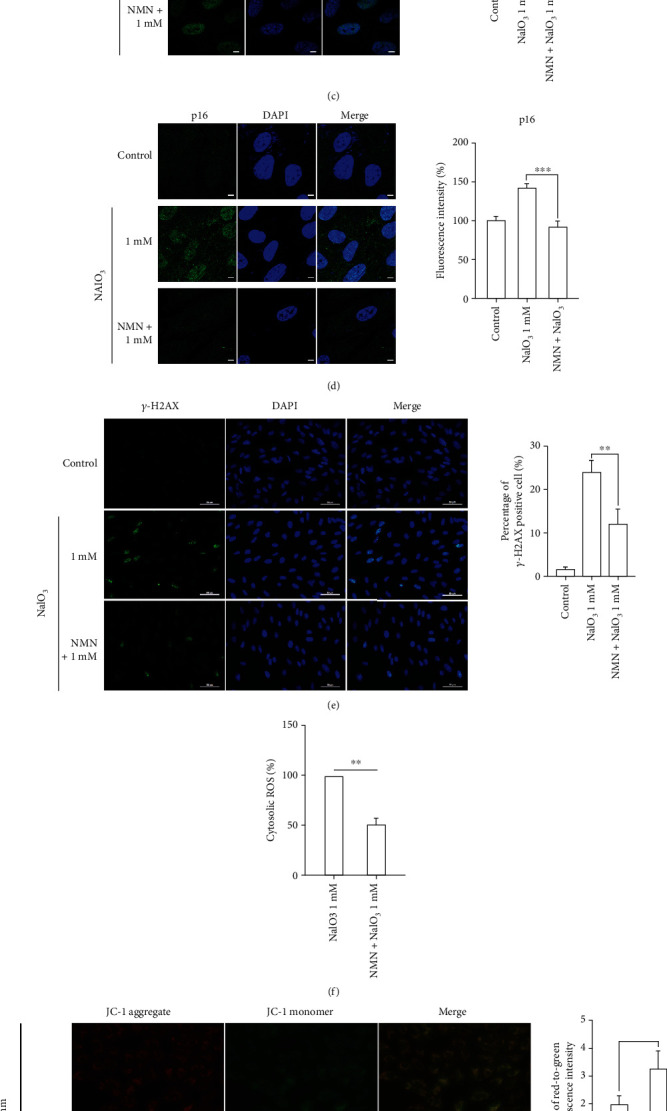
(a) Cellular NAD+ levels in ARPE-19 cells. The cells were treated with various concentrations of NMN for 24 h prior to 1 mM NaIO_3_ treatment for 48 h (*n* = 3). (b) The representative images and the quantitative results of SA-*β*-gal staining. The cells were challenged by 1 mM NaIO_3_ for 48 h with or without 1000 *μ*M NMN pretreatment (*n* = 3). (c–d) Representative images of immunofluorescence assay and mean fluorescence intensities of p21 and p16 in APRE-19 cells. Cells were treated with 1 mM NaIO_3_ for 48 hr with or without 1000 *μ*M NMN pretreatment. The pictures were taken by confocal microscope; scale bar = 10 *μ*m (*n* = 3). (e) The *λ*-H2AX immunostaining was performed to show the DNA damage level in ARPE-19 cells treated as indicated; scale bar = 50 *μ*m. The DNA damage level was reflected by the average number of *λ*-H2AX-positive cells (*n* = 3). (f) The cytosolic ROS levels were revealed by H2DCFDA staining, and the results were obtained by flow cytometry (*n* = 3). (g) MMP in ARPE-19 cells with indicated treatment was revealed by JC-1 staining, and the results were obtained by fluorescent microscopy. The red-to-green fluorescence intensity was used to quantitatively evaluate the mitochondrial potential; scale bar = 50 *μ*m (*n* = 3). (h) The cellular ATP levels in ARPE-19 cells in the indicated groups (*n* = 5). Data represent mean ± SD of at least three independent experiments, ∗*p* < 0.05, compared versus control, ∗∗*p* < 0.01, ∗∗∗*p* < 0.001 compared versus the indicated groups.

Because NAD^+^ plays a prominent role in the aging process and we observed in this study that NAD^+^ levels decreased in senescent RPE cells, we speculated that NAD^+^ supplementation could ameliorate cellular senescence. To prove this, we treated ARPE-19 cells with various concentrations of NMN for 24 h before NaIO_3_ treatment. The results showed that supplementation with 800 and 1000 *μ*M NMN adequately restored cellular NAD^+^ levels (Figure 5(a)). Then, we detected the expression of three canonical senescence markers, and all of them had a significantly reduced expression following pretreatment with 1000 *μ*M NMN (Figures 5(b)–5(d)). The number of *λ*-H2AX-positive cells were also reduced by 50% after NMN administration (Figure 5(e)). The cytosolic ROS levels in the NMN group were significantly lower than those in the NaIO_3_ group (Figure 5(f)), indicating that NMN could prevent ARPE-19 cells from oxidative stress-related DNA damage. The restoration of MMP and cellular ATP levels indicated that NMN treatment could protect RPE cells from NaIO_3_-induced mitochondrial damage (Figures 5(g) and 5(h)).

### 3.5. Sirt1 Homeostasis Is Essential in Mediating the Antisenescence Function of NAD+ Supplementation

**Figure 6:**
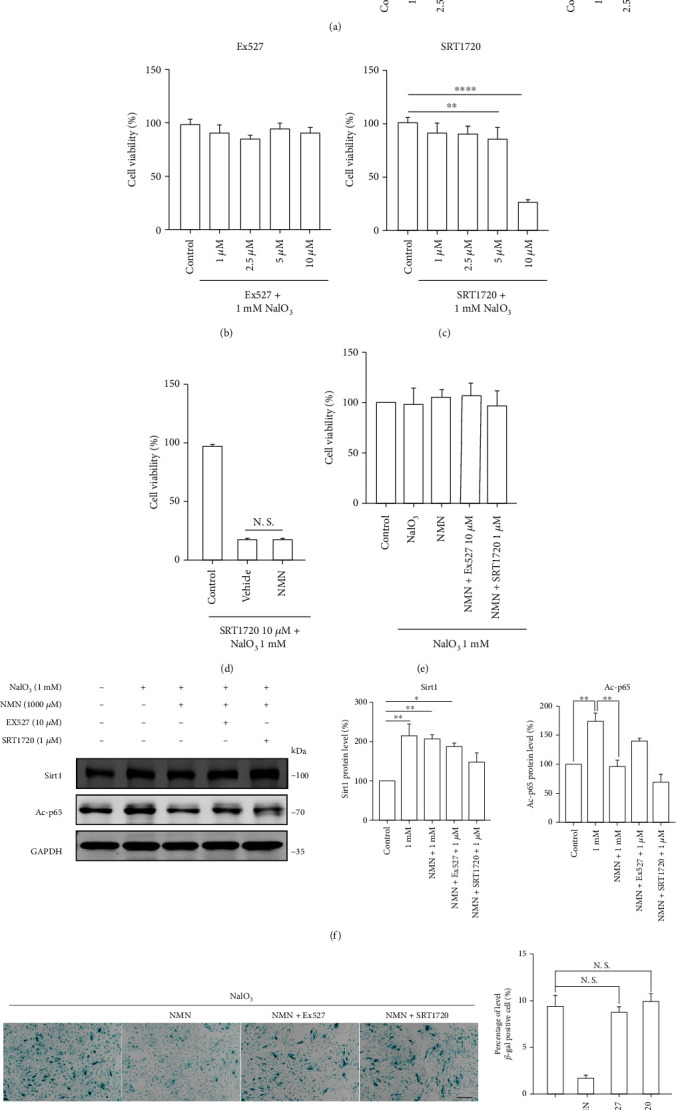
(a) The levels of Sirt1 and Acetyl-p65 proteins and the statistical results in senescent ARPE-19 cells. (b–c) Cell viability of ARPE-19 cells. The cells were pretreated with the indicated concentrations of Ex527 and SRT1720 for 24 h prior to treatment with 1 mM NaIO_3_ for 48 h (*n* = 6). (d) The results of cell viability in cells co-cultured with NMN (1000 *μ*M) and SRT1720 (10 *μ*M) or with vehicle and SRT1720 (10 *μ*M) for 24 h prior to treatment with 1 mM NaIO_3_ for 48 h (*n* = 3). (e–g) The ARPE-19 cells were pretreated with NMN, NMN, and Ex527 or NMN and SRT1720 at the indicated concentrations for 24 h prior to treatment with 1 mM NaIO_3_ for 48 h. (e) Cell viability was detected by CCK-8 assay (*n* = 3). (f) The levels of Sirt1 and Acetyl-p65 proteins in ARPE-19 cells with the indicated treatment were determined by western blot analysis (*n* = 3). (g) The representative images and statistical results of SA-*β*-gal staining assay; scale bar = 200 *μ*m (*n* = 3). Data represent mean ± SD of at least three independent experiments, ∗*p* < 0.05, ∗∗*p* < 0.01, ∗∗∗*p* < 0.001, ∗∗∗∗*p* < 0.0001, and the *p* values represented the statistical significance of comparisons versus control except for additional labeling in the figures.

Sirt1 is a vital enzyme of sirtuins that functions through the consumption of cellular NAD^+^. Previous studies have demonstrated that NMN enhances hepatic insulin sensitivity partly through Sirt1 activation [30]. Thus, we decided to explore whether Sirt1 was an essential factor for the antisenescence effects of NMN. First, we determined the expression of Sirt1 and acetyl-p65 and found that the protein levels of Sirt1 were upregulated, whereas the activity of Sirt1 remained inhibited in the senescent cells (Figure 6(a)). We then speculated that this phenomenon might be ascribed to the decreased NAD^+^ levels in these cells. Next, we modulated the activity of Sirt1 by using its agonist (SRT1720) and inhibitor (Ex527). To determine an optimal concentration for cells to remain viable, we assessed the cell viability of ARPE-19 cells after treatment with SRT1720, and we observed an unexpected and massive cell death when the concentration of SRT1720 reached 10 *μ*M (Figures 6(b) and 6(c)). Moreover, this effect was not due to NAD^+^ depletion (Figure 6(d)). Therefore, we chose a relatively smaller dose of SRT1720 to reduce the side effects previously observed (Figure 6(e)). The protein levels of Sirt1 and acetyl-p65 confirmed the effect of SRT1720 and Ex527, indicating that NMN treatment could significantly activate Sirt1 (Figure 6(f)). So, we subsequently performed SA-*β*-gal analysis to verify whether Sirt1 was a crucial factor in mediating the effects of NMN. The result revealed that both Sirt1 inhibition and activation blunted the antisenescence effect of NMN (Figure 6(g)).

**Figure 7:**
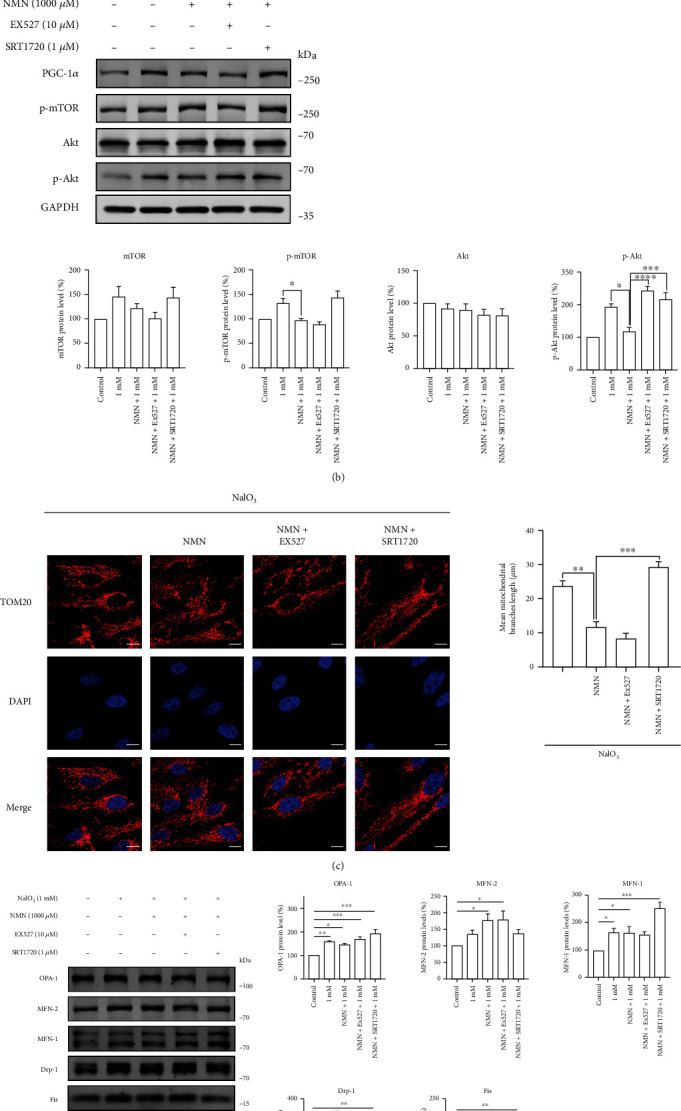
(a–d) The ARPE-19 cells were pretreated with NMN, NMN, and Ex527 or NMN and SRT1720 at the indicated concentrations for 24 h prior to treatment with 1 mM NaIO_3_ for 48 h. (a–b) Protein levels and statistical results of the indicated proteins in ARPE-19 cells. GAPDH or *β*-tubulin was used as loading control (*n* = 3). (c) Representative images of immunofluorescence of Tom20 in ARPE-19 cells; scale bar = 10 *μ*m (*n* = 3). (d) Protein levels of mitochondrial dynamic proteins in ARPE-19 cells. GAPDH was used as loading control (*n* = 3). Data represent mean ± SD of at least three independent experiments, ∗*p* < 0.05, ∗∗*p* < 0.01, ∗∗∗*p* < 0.001, ∗∗∗∗*p* < 0.0001, and the *p* values represented the statistical significance of comparisons versus control except for additional labeling in the figures.

To identify why SRT1720 activated Sirt1 but failed to recapitulate the antisenescence effects of NMN, we studied the key pathways that are altered in senescent RPE cells (Figures 7(a) and 7(b)). The NMN-treated group showed lower levels of PGC-1*α* and p-AMPK activation, which might be due to the decreased mitochondrial injury observed in that group. However, the SRT1720 + NMN group had higher protein levels of PGC-1*α* (1.36-fold) and p-AMPK (1.85-fold) than those in the NMN group. The downregulation of Akt/mTOR signaling in the NMN-treated group was ascribed to the observed reduction in the rate of *λ*-H2AX positivity. Meanwhile, the Sirt1 activation or inhibition group failed to mitigate Akt activation. Moreover, the results of the mitochondrial morphology analysis and the expression levels of mitochondrial dynamic proteins indicated that SRT1720 treatment caused a significant upregulation in the levels of OPA-1 and MFN-1 and mitochondria hyperfusion, which might be associated with Sirt1 overactivation-related senescence (Figures 7(c) and 7(d)). In conclusion, our results suggested that Sirt1 activation is required for the antisenescence effects of NMN, but the overactivation of Sirt1 could cause additional cellular stress and decrease the effects of NMN treatment.

### 3.6. NMN Decreases RPE Dysfunction and Senescence-Associated Inflammation

**Figure 8:**
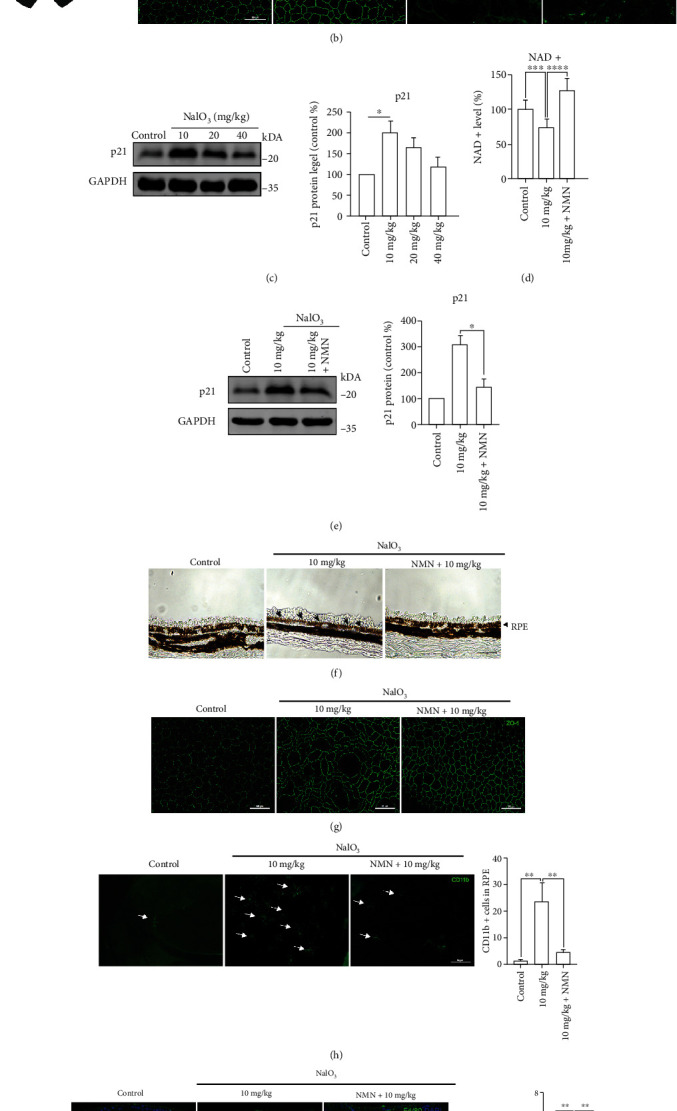
(a) H&E staining of retinal sections from mice at 7 days after 0-80 mg/kg NaIO_3_ injection; scale bar = 50 *μ*m (*n* = 3). (b) ZO-1 staining of the RPE flatmounts from mice treated with 0-40 mg/kg NaIO_3_ for 7 days; scale bar = 50 *μ*m (*n* = 3). (c) p21 protein levels were detected in the RPE-choroid complex from mice in the corresponding groups. GAPDH was used as loading control; compared versus control (*n* = 3). (d) NAD^+^ levels in RPE-choroid complex of mice under the indicated administration (*n* = 3). (e) p21 protein levels in the RPE-choroid complex from mice treated as indicated (*n* = 3). (f) Representative images of SA-*β*-gal staining in retinal sections from mice in the indicated groups; the SA-*β*-gal-positive RPE cells were indicated by arrows; scale bar = 50 *μ*m (*n* = 3). (g) Flatmount ZO-1 staining in the RPE layer of mice treated with the indicated drugs; scale bar = 50 *μ*m (*n* = 3). (h) CD11b staining was performed on the RPE flatmounts from mice treated as indicated; the CD11b^+^ cells were noted by arrows; scale bar = 50 *μ*m (*n* = 3). (i) F4/80 staining was performed on the retinal slices from mice treated as indicated. F4/80^+^ cells were noted by arrows. Scale bar = 50 *μ*m (*n* = 3). Data represent mean ± SD of at least three independent experiments; ∗*p* < 0.05, ∗∗*p* < 0.01, ∗∗∗*p* < 0.001, ∗∗∗∗*p* < 0.0001, and the *p* values represented the statistical significance of comparisons versus control except for additional labeling in the figures; ONL: outer nuclear layer; RPE: retinal pigment epithelium.

Since the ARPE-19 cell line has some limitations on mimicking the physiological characteristics of the RPE cells, we evaluated whether NMN could exert antisenescence effects *in vivo*. We established an animal model of RPE senescence by administering an intraperitoneal injection of NaIO_3_ to C57BL/6J mice. Our results showed that dosages above 40 mg/kg caused severe damage to the RPE and a thinning of ONL, and thus, these doses were not suitable to establish a senescence model (Figure 8(a)). Therefore, we performed ZO-1 staining on the RPE flatmounts and detected the levels of p21 protein of RPE to determine the suitable concentration for further investigation. Our results showed that treatment with NaIO_3_ at a concentration of 10 mg/kg could induce morphologic changes in the RPE and increase p21 protein levels (Figures 8(b) and 8(c)). Subsequently, we pretreated the mice through an intraperitoneal injection of NMN to explore the function of NMN *in vivo*. The NAD^+^ levels in the RPE were reduced after treatment with NaIO_3_, while they were significantly restored upon NMN treatment (Figure 8(d)). The decreased protein levels of p21 and the decline of positive rate of SA-*β*-gal staining indicated that NMN suppressed the effects of NaIO_3_-induced senescence (Figures 8(e) and 8(f)). Results of the immunofluorescence assay showed that the structure of the RPE layer was more regular in the NMN-pretreated group than in the vehicle-pretreated group (Figure 8(g)). An important adverse effect linking senescence to age-related disease is the potential of senescent cells to recruit immune cells to the resident tissue and cause local inflammation [31]. Thus, we immunostained the inflammatory cells with CD11b and F4/80 in the subretinal space of the RPE flatmounts and tissue sections. We demonstrated that the CD11b^+^ and F4/80^+^ cells could be recruited to the subretinal space by senescent RPE cells and that this process was prevented by pretreatment with NMN (Figures 8(h) and 8(i)).

## 4. Discussion

NAD^+^ is an important metabolic cofactor that controls metabolism and energy homeostasis in organisms. Recent studies demonstrated that NAD^+^ plays a crucial role in cellular and tissue senescence [32]. Studies on C57BL/6J mice (2–18 months) showed that the NAD^+^ levels of primary RPE cells declined significantly with age [33]. Because of the lack of an optimal acute experimental model, that study used FK866 to mimic the NAD^+^ decline and to study the senescence process in the RPE. However, FK866 treatment decreased NAD^+^ levels but failed to cause changes related to AMD. Therefore, there is an urgent need to establish an optimal senescence-associated AMD model. NaIO_3_ is widely used for mimicking the characteristics of dry AMD [16, 28]; however, it is rarely used to study senescence. In this study, we observed that NaIO_3_ treatment depleted cellular NAD^+^ levels and induced RPE senescence both *in vivo* and *in vitro*. Cell/animal models of senescence acquired some pre-AMD changes such as RPE dysfunction and immune cell infiltration [34].

NaIO_3_ is commonly used as an oxidant. Although oxidative stress is a canonical initiator of senescence, our results strongly indicated that mitochondrial changes (mitochondrial dysfunction, mitochondrial dynamic changes, and mitochondrial biogenesis) were more involved in NaIO_3_-induced senescence. Mitochondrial dysfunction is known to be correlated with RPE degeneration and the onset of AMD [27]. To date, however, a very small number of studies have focused on mitochondrial dysfunction in relation to RPE senescence in AMD. The results from our study facilitated in bridging this gap and proposed a novel pathway for the pathogenesis of AMD.

Ebeling et al. proposed that NMN treatment could increase ATP production in the RPE from donors with AMD [35]. Our results not only confirmed this but also demonstrated that NMN treatment eliminated senescent RPE cells, relieved the morphological abnormality of RPE, and alleviated subretinal inflammation. The tight junctions between the RPE cells enable the RPE to form the outer blood-retinal barrier (BRB), which is one of the crucial physiological functions of the RPE [7]. In the later stages of AMD, the loss of the BRB due to the death of RPE cells accelerates the dysfunction of the photoreceptors. In our study, the senescent RPE showed an attenuated barrier function due to the loss of their typical hexagonal structure. Pretreatment with NMN significantly mitigated the loss of senescence-related barrier function. However, it should be noted that all these changes were independent of RPE cell death, indicating that NMN supplementation might be a potential therapeutic strategy for pre-AMD changes.

Sirt1 is well-known for sustaining genome stability and programming the stress-response genes [36, 37]. All these functions seemed to endow Sirt1 with an antiaging role. However, previous reports on the pharmacological activation of Sirt1 were still in debate since both beneficial and lethal effects were reported [38, 39]. In our study, we noticed that high concentrations of SRT1720 were lethal to ARPE-19 cells exposed to NaIO_3_. Meanwhile, the activation of Sirt1 is required for the antisenescence effects of NMN since Ex527 could blunt the effects of NMN due to the inhibition of Sirt1 activity. It is noteworthy that Sirt1 overactivation by SRT1720 could induce additional cellular stress, which was reflected by the activation of AMPK and Akt signalling. Because cells pretreated with SRT1720 showed hyperfused mitochondria similar to the cells treated with NaIO_3_ only, we then speculated that the overactivation of Sirt1 could aggravate NaIO_3_-related mitochondrial injury. In summary, our results indicated that undisturbed Sirt1 function is required for the antisenescence effects of NMN. Likewise, we suggested that the beneficial effects of Sirt1 overactivation should be interpreted prudently. Compared to SRT1720, NMN exhibited a more physiological way because Sirt1 activation induced by NMN was based on the increase in NAD^+^ levels.

Previous studies have shown that the mTOR signalling plays a role in cellular senescence and the repression of mTORC1 could prevent RPE senescence [40, 41]. Moreover, previous RNA-Seq analyses based on human AMD donors showed an overactivation of the AMPK and mTOR pathway in RPE cells [42, 43]. Since both AMPK and Akt/mTOR signalling were activated in our senescent cells, our results highly suggest that the two pathways are essential in linking cellular senescence to the onset of AMD. This potential connection is reemphasized by NMN because NMN decreased the pre-AMD changes through inhibiting the activation of AMPK and Akt/mTOR signalling.

## 5. Conclusion

The present study demonstrated that NMN treatment could ameliorate the cellular senescence and retinal inflammation caused by NaIO_3_ exposure. This protective effect was executed upon NAD^+^ supplementation, which enhanced mitochondrial function, reduced DNA damage, and suppressed the activation of AMPK and Akt/mTOR signalling. NMN treatment also maintained the hexagonal structure of RPE *in vivo* and reduced senescence-related retinal inflammation. Moreover, undisturbed Sirt1 activity was required for NMN to exert its antisenescent effects. Overall, our results provided novel insights into the pathogenesis of AMD and a potential therapeutic strategy for AMD from the perspective of cellular and histological senescence.

## Data Availability

The datasets used and/or analyzed during the current study are available from the corresponding author on reasonable request.
